# Changing Threshold-Based Segmentation Has No Relevant Impact on Semi-Quantification in the Context of Structured Reporting for PSMA-PET/CT

**DOI:** 10.3390/cancers14020270

**Published:** 2022-01-06

**Authors:** Patrick W. Mihatsch, Matthias Beissert, Martin G. Pomper, Thorsten A. Bley, Anna K. Seitz, Hubert Kübler, Andreas K. Buck, Steven P. Rowe, Sebastian E. Serfling, Philipp E. Hartrampf, Rudolf A. Werner

**Affiliations:** 1 Department of Diagnostic and Interventional Radiology, University Hospital of Würzburg, 97080 Würzburg, Germany; beissert_m@ukw.de (M.B.); bley_t@ukw.de (T.A.B.); 2 The Russell H. Morgan Department of Radiology and Radiological Science, Division of Nuclear Medicine and Molecular Imaging, Johns Hopkins University School of Medicine, Baltimore, MD 21287, USA; mpomper@jhmi.edu (M.G.P.); srowe8@jhmi.edu (S.P.R.); 3 Department of Urology and Paediatric Urology, University Hospital of Würzburg, 97080 Würzburg, Germany; seitz_a3@ukw.de (A.K.S.); kuebler_h@ukw.de (H.K.); 4 Department of Nuclear Medicine, University Hospital of Würzburg, 97080 Würzburg, Germany; buck_a@ukw.de (A.K.B.); serfling_s1@ukw.de (S.E.S.); hartrampf_p@ukw.de (P.E.H.); werner_r1@ukw.de (R.A.W.)

**Keywords:** ^18^F-PSMA-1007, PET/CT, staging, prostate cancer, standardized reporting system, PSMA-RADS

## Abstract

**Simple Summary:**

Molecular imaging of patients with prostate cancer is widely utilized. We aimed to determine whether changes in post-processing parameters, such as maximum intensity thresholds, can significantly alter results. We investigated 623 lesions that were positive on a molecular imaging scan and could not find any relevant impact on results when certain parameters were changed, in particular in lesions indicative for metastases of prostate cancer.

**Abstract:**

Prostate-specific membrane antigen (PSMA)-directed positron emission tomography/computed tomography (PET/CT) is increasingly utilized for staging of men with prostate cancer (PC). To increase interpretive certainty, the standardized PSMA reporting and data system (RADS) has been proposed. Using PSMA-RADS, we characterized lesions in 18 patients imaged with ^18^F-PSMA-1007 PET/CT for primary staging and determined the stability of semi-quantitative parameters. Six hundred twenty-three lesions were categorized according to PSMA-RADS and manually segmented. In this context, PSMA-RADS-3A (soft-tissue) or -3B (bone) lesions are defined as being indeterminate for the presence of PC. For PMSA-RADS-4 and -5 lesions; however, PC is highly likely or almost certainly present [with further distinction based on absence (PSMA-RADS-4) or presence (PSMA-RADS-5) of correlative findings on CT]. Standardized uptake values (SUV_max_, SUV_peak_, SUV_mean_) were recorded, and volumetric parameters [PSMA-derived tumor volume (PSMA-TV); total lesion PSMA (TL-PSMA)] were determined using different maximum intensity thresholds (MIT) (40 vs. 45 vs. 50%). SUV_max_ was significantly higher in PSMA-RADS-5 lesions compared to all other PSMA-RADS categories (*p* ≤ 0.0322). In particular, the clinically challenging PSMA-RADS-3A lesions showed significantly lower SUV_max_ and SUV_peak_ compared to the entire PSMA-RADS-4 or -5 cohort (*p* < 0.0001), while for PSMA-RADS-3B this only applies when compared to the entire PSMA-RADS-5 cohort (*p* < 0.0001), but not to the PSMA-RADS-4 cohort (SUV_max_, *p* = 0.07; SUV_peak_, *p* = 0.08). SUV_mean_ (*p* = 0.30) and TL-PSMA (*p* = 0.16) in PSMA-RADS-5 lesions were not influenced by changing the MIT, while PSMA-TV showed significant differences when comparing 40 vs. 50% MIT (*p* = 0.0066), which was driven by lymph nodes (*p* = 0.0239), but not bone lesions (*p* = 0.15). SUV_max_ was significantly higher in PSMA-RADS-5 lesions compared to all other PSMA-RADS categories in ^18^F-PSMA-1007 PET/CT. As such, the latter parameter may assist the interpreting molecular imaging specialist in assigning the correct PSMA-RADS score to sites of disease, thereby increasing diagnostic certainty. In addition, changes of the MIT in PSMA-RADS-5 lesions had no significant impact on SUV_mean_ and TL-PSMA in contrast to PSMA-TV.

## 1. Introduction

Prostate cancer (PC) is the second most frequent cancer in men, with more than 1.4 million new cases worldwide in 2020 [1]. The introduction of imaging agents targeting prostate-specific membrane antigen (PSMA), a type II transmembrane glycoprotein that is overexpressed on PC cells [2,3], has further improved the ability to detect and ascertain the presence of metastatic disease compared to conventional imaging [4], showing high diagnostic accuracy for primary staging and restaging during biochemical recurrence [5,6,7]. Several PSMA-targeted positron emission tomography (PET) radioligands, especially ^68^Ga-labeled compounds, have been established for clinical use [8]. ^18^F-labeled radiotracers, such as ^18^F-PSMA-1007, offer several key advantages when compared to their ^68^Ga-labeled counterparts, most notably a longer half-life (110 min vs. 68 min), improved spatial resolution, and decreased imaging noise (due to lower positron energy) as well as potential cost savings due to centralized cyclotron-production with dispatch to remote PET centers [5,8,9].

To standardize interpretation in patients imaged with PSMA-directed radiotracers, we proposed a structured reporting and data system (PSMA-RADS version 1.0) [10]. Based on a 5-point scale, this framework categorizes lesions based on their likelihood of PC involvement, with PSMA-RADS-1 being certainly benign, whereas PC is almost certainly present in a PSMA-RADS-5 lesion [10,11,12], thereby allowing for lesion-level characterization that may be helpful in planning focal therapy for limited volume disease and defining work-up strategies for indeterminate findings.

To date, PSMA-RADS has been validated in various contexts using the ^18^F-labeled PSMA imaging agent ^18^F-DCFPyL as well as ^68^Ga-PSMA-11 [12,13,14], but has not been valudated for [^18^F]PSMA-1007 positron emission tomography/computed tomography (PET/CT) scans. Given substantial differences in the lesion detection rate and normal biodistribution of those radiotracers [8,15,16], we aimed to examine the use of PSMA-RADS for ^18^F-PSMA-1007. In addition, we aimed to determine the stability of PSMA-RADS-based semi-quantification when different maximum intensity thresholds (MIT) were applied, in particular for PSMA-RADS-5 lesions (i.e., PC almost certainly present).

## 2. Materials and Methods

### 2.1. Patient Population

This observational retrospective study was approved by the local Institutional Review Board (IRB-Nr.: 20210204 03; date of approval: 23 March 2021) and conducted in accordance with the ethical standards of the institutional and/or national research committee as well as with the 1964 Declaration of Helsinki and its later amendments. An institutional database search for the time period from December 2018 to January 2021 identified 31 patients with newly diagnosed PC who underwent ^18^F-PSMA-1007 PET/CT imaging for primary staging. Patients with concomitant malignancies (n = 1), unavailability of histopathology (n = 3) and/or prior initiation of a systemic treatment, such as androgen deprivation therapy (ADT; n = 9) were excluded in order to exclude potential influences, e.g., of medication, on the level of PSMA expression. Hence, a total of 18 patients with histologically proven and treatment-naïve PC at date of scan were eligible for this study (Table 1).

### 2.2. Preparation of ^18^F-PSMA-1007

Radiosynthesis of ^18^F-PSMA-1007 (radiochemical purity: ≥95%) was carried out using a GE TRACERlab MX synthesis module (GE Medical Systems, Uppsala, Sweden) as described in [17].

### 2.3. Image Acquisition and Reconstruction

^18^F-PSMA-1007 PET/CTs were performed within 28 ± 14 days after initial histopathological diagnosis. As per usual practice at our institution, patients were asked to be *nil per os* (excluding water and medications) for a minimum of 4 h prior to the examination. Patients received an i.v. injection of the radiotracer ^18^F-PSMA-1007 with a mean activity of 301 ± 15 MBq, followed by oral ingestion of an iodine-based contrast agent (30 mL Peritrast^®^ in 1 L of water). Directly prior to the scan, patients were asked to void their urinary bladders. Furosemide was not administered. After a mean radiotracer uptake period of 91 ± 10 min, whole body imaging (from mid-thighs to skull base, in a supine position) was performed with either a 64-detector PET/CT scanner (Siemens Biograph mCT 64, Siemens Healthineers AG, Erlangen, Germany) or a 128-detector PET/CT scanner (Siemens Biograph mCT Flow 128 Edge, Siemens Healthineers AG, Knoxville, TN, USA). All PET emission data were acquired in three-dimensional mode using 6–8 bed positions (depending on the patient’s height) with a scan duration of 2 min per bed position [18]. Subsequently, after i.v. injection of an iodine-based contrast agent (1 mL/kg body weight of Imeron^®^ 350), a monophasic full-dose CT scan (100–120 kV, mA modulated) was performed for anatomic correlation and attenuation correction [18,19]. Iterative image reconstruction was achieved using an ordered subset expectation maximization (OSEM) algorithm either with 3 iterations and 24 subsets followed by post-reconstruction Gaussian filter smoothing (full width at half maximum (FWHM): 2 mm; Siemens TrueX; Siemens Biograph mCT 64) or with 3 iterations and 21 subsets followed by post-reconstruction Gaussian filter smoothing (FWHM: 2 mm; Siemens TrueX + TOF ultraHD; Siemens Biograph mCT Flow 128 Edge) [19]. Reconstructed PET images had a spatial resolution with an FWHM of 4.7 mm (Siemens Biograph mCT Flow 128 Edge) or 5.7 mm (Siemens Biograph mCT 64) [19].

### 2.4. Image Analysis

PET, CT, and hybrid PET/CT overlay images were assessed using a dedicated workstation equipped with syngo.via imaging software, version VB50B_HF02 (Siemens Healthineers AG, Erlangen, Germany). Scans were evaluated by a reader with 3 years’ experience in reading PSMA-targeted PET/CTs (P.W.M.) who previously underwent a training session for the standardized reporting system PSMA-RADS version 1.0 [10]. In ambiguous cases, PSMA-RADS categories were assigned in a consensus read with two experienced readers, including a board-certified radiologist and nuclear medicine physician (M.B., R.A.W.).

All measurable lesions were noted along with their location (bone, lymph node, liver, lung, soft tissue, primary) and were assigned to a PSMA-RADS category [14] (with soft tissue lesions defined as lesions not being assigned to one of the other five compartments). In total, four patients (4/18, 22.2%) with disseminated skeletal metastatic disease were encountered, in whom up to ten separate bone lesions were recorded. In brief, PSMA-RADS-1A and -1B represent definitely benign lesions, while likely benign lesions are categorized as PSMA-RADS-2. By contrast, lesions in which the presence of PC is highly likely or almost certainly present are classified as PSMA-RADS-4 or PSMA-RADS-5, with further distinction based on absence (PSMA-RADS-4) or presence (PSMA-RADS-5) of correlative findings on computed tomography (CT) [10,11]. PSMA-RADS-3, however, is the most complex category, and is divided into four subcategories [10]. PSMA-RADS-3A and PSMA-RADS-3B define lesions that would be typical for PC, but having only subtle radiotracer uptake in soft tissue or bone lesions, respectively, rendering those findings equivocal. PSMA-RADS-3C and PSMA-RADS-3D lesions, however, are unlikely to represent PC and require further work-up [10].

By using a 3-dimensional segmentation method, an isocontour volume of interest (VOI) was created separately for all lesions before the maximum and peak standardized uptake value corrected to body weight (SUV_max_ and SUV_peak_) were recorded for each lesion [20,21]. While keeping the VOI for each lesion identical, different MITs were then applied (40, 45, and 50%), i.e., a pre-specified percentage VOI which only includes all voxels equal to or above the chosen percentage of SUV_max_ [22]. In addition, the mean standardized uptake value (SUV_mean_) and volumetric parameters were measured for each lesion, yielding a PSMA-derived tumor volume (PSMA-TV, cm^3^) and total lesion PSMA (TL-PSMA, cm^3^). As SUV_mean_, PSMA-TV and TL-PSMA are influenced by changes of the MIT, all lesions were measured applying a MIT of 40, 45, and 50% in the identical VOI, as described for ^68^Ga-labeled PSMA PET in [20]. TL-PSMA was calculated as follows [20]:

PSMA-TV × SUV_mean_ (1)

As such, for those three parameters, values for MIT of 40, 45, and 50% were recorded.

### 2.5. Statistical Analysis

Statistical analyses were performed with GraphPad Prism software, version 9.2.0.332 (GraphPad Software, Inc., San Diego, CA, USA) and Microsoft Excel 2016 (Microsoft Corporation, Redmond, WA, USA). All continuous data are expressed as either mean or median value ± standard deviation (SD) and range in parentheses. Categorical variables are presented as frequency and percentage in parentheses. Comparisons between subgroups were made using the Kruskal-Wallis test. *p* values < 0.05 were considered statistically significant (*, *p* < 0.05; **, *p* < 0.01; ***, *p* < 0.001; ****, *p* < 0.0001).

## 3. Results

### 3.1. Prevalence of PSMA-RADS-4 and -5 Lesions Was High

Across all patients, a total of 623 lesions were analyzed on ^18^F-PSMA-1007 PET/CT, with the vast majority of lesions either located in lymph nodes (449/623, 72.1%) or in the skeleton (105/623, 16.8%). Moreover, the following compartments were also assessed: lung (28/623, 4.5%), soft tissue (20/623, 3.2%), primary (18/623, 2.9%), and liver lesions (3/623, 0.5%).

**Figure 1:**
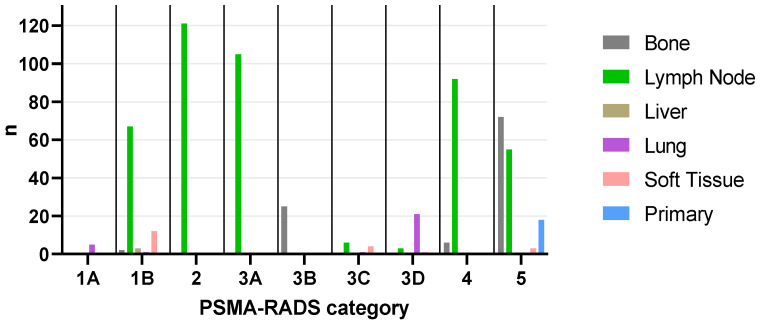
Bar graph showing the distribution of total lesions (n = 623) across all compartments and PSMA-RADS categories. Bone: 1B (n = 2), 3B (n = 25), 4 (n = 6), 5 (n = 72), total (n = 105); Lymph Node: 1B (n = 67), 2 (n = 121), 3A (n = 105), 3C (n = 6), 3D (n = 3), 4 (n = 92), 5 (n = 55), total (n = 449); Liver: 1B (n = 3), total (n = 3); Lung: 1A (n = 5), 1B (n = 1), 3C (n = 1), 3D (n = 21), total (n = 28); Soft Tissue: 1B (n = 12), 3C (n = 4), 3D (n = 1), 5 (n = 3), total (n = 20); Primary: 5 (n = 18), total (n = 18). PSMA-RADS = prostate-specific membrane antigen reporting and data system.

A plurality of lesions were categorized as PSMA-RADS-5 (148/623, 23.8%), followed by PSMA-RADS-2 (121/623, 19.4%), PSMA-RADS-3A (105/623, 16.9%), PSMA-RADS-4 (98/623, 15.7%), PSMA-RADS-1B (85/623, 13.6%), PSMA-RADS-3B (25/623, 4.0%), PSMA-RADS-3D (25/623, 4.0%), PSMA-RADS-3C (11/623, 1.8%), and PSMA-RADS-1A (5/623, 0.8%). Sorted by PSMA-RADS categories, the distribution among organ compartments can be found in Table A1, while an overview for each patient is given in Table A2. The distribution of all lesions (n = 623) across all compartments and PSMA-RADS categories is shown in Figure 1.

### 3.2. SUV_max_ of PSMA-RADS-5 Lesions Differed Significantly When Compared to All Other PSMA-RADS Categories

Including all lesions (independent of organ compartments), lesions classified as PSMA-RADS-1A, -1B, -2, and -3D each demonstrated a significantly lower SUV_max_ and SUV_peak_ when compared to PSMA-RADS-4 (SUV_max_, *p* < 0.0001; SUV_peak_, *p* ≤ 0.0002) or PSMA-RADS-5 (SUV_max_, *p* < 0.0001; SUV_peak_, *p* < 0.0001). Interestingly, lesions classified as PSMA-RADS-3A also showed a significantly lower SUV_max_ and SUV_peak_ compared to PSMA-RADS-4 or -5 (*p* < 0.0001). For all lesions categorized as PSMA-RADS-3C, SUV_max_ was significantly lower compared to PSMA-RADS-5 (*p* = 0.0322); however, no significant difference was noted for SUV_max_ compared to PSMA-RADS-4 (*p* > 0.99) as well as for SUV_peak_ compared to both PSMA-RADS-4 (*p* > 0.99) and -5 (*p* = 0.08). Lesions categorized as PSMA-RADS-4 also showed a significantly lower SUV_max_ (*p* = 0.0027) and SUV_peak_ (*p* < 0.0001) compared to PSMA-RADS-5.

Lymph nodes categorized as PSMA-RADS-1B, -2, and -3A showed a significantly lower SUV_max_ (*p* < 0.0001) and SUV_peak_ (*p* < 0.0001) compared to the entire PSMA-RADS-4 or -5 cohort. Lymph nodes in category PSMA-RADS-3D (n = 3) also showed a significantly lower SUV_max_ compared to PSMA-RADS-5 (*p* = 0.0131), but not -4 (*p* = 0.28), while SUV_peak_ did not significantly differ (*p* ≥ 0.16). For PSMA-RADS-3C (n = 6), SUV_max_ and SUV_peak_ did not significantly differ compared to PSMA-RADS-4 (*p* > 0.99) or -5 (SUV_max_, *p* = 0.17; SUV_peak_, *p* = 0.29). Lymph nodes classified as PSMA-RADS-4 showed a significantly lower SUV_max_ (*p* = 0.0007) and SUV_peak_ (*p* < 0.0001) compared to the entire PSMA-RADS-5 cohort.

For bone lesions, significantly lower SUV_max_ and SUV_peak_ for PSMA-RADS-3B lesions were noted when compared to the entire PSMA-RADS-5 cohort (*p* < 0.0001), but not compared to the PSMA-RADS-4 cohort (SUV_max_, *p* = 0.07; SUV_peak_, *p* = 0.08). SUV_max_ and SUV_peak_ of PSMA-RADS-4 bone lesions did not significantly differ from the entire PSMA-RADS-5 cohort (*p* ≥ 0.62).

**Figure 2:**
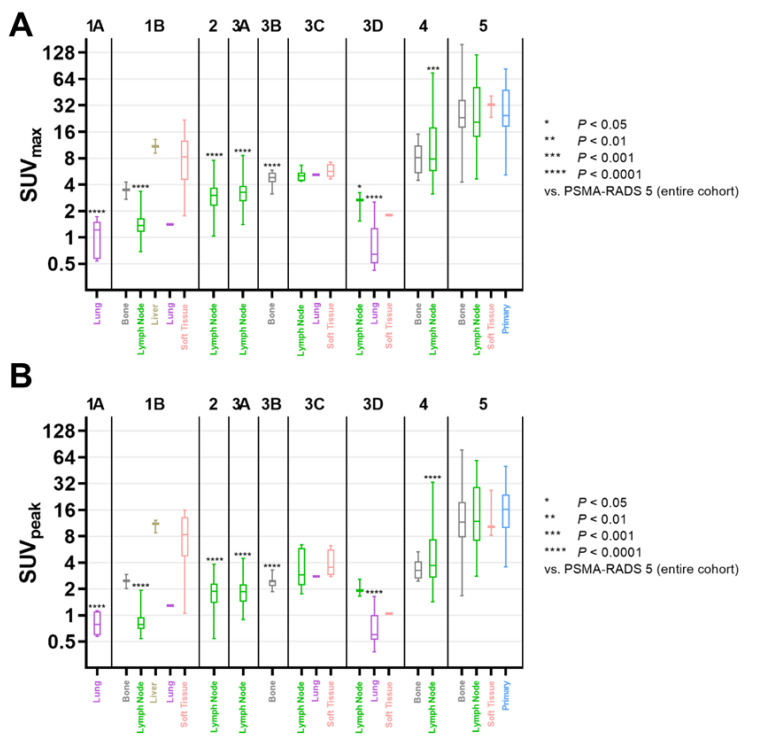
Box-and-whisker charts showing the maximum (SUV_max_, (**A**)) and peak standardized uptake value (SUV_peak_, (**B**)) among compartments and different PSMA-RADS categories. For each subgroup of PSMA-RADS categories 1-4, mean values were compared to PSMA-RADS-5. Both SUV_max_ and SUV_peak_ of lymph nodes in PSMA-RADS categories 1B, 2, 3A, and 4 differed significantly compared to PSMA-RADS-5 lesions (SUV_max_, *p* ≤ 0.0007; SUV_peak_, *p* < 0.0001); lymph nodes in PSMA-RADS-3D also showed a significantly lower SUV_max_ (*p* = 0.0131). Regarding bone lesions, compared to the entire PSMA-RADS-5 category SUV_max_ and SUV_peak_ were significantly lower only for PSMA-RADS-3B (*p* < 0.0001), but not for PSMA-RADS categories 1B (*p* ≥ 0.49) and 4 (*p* ≥ 0.62). Data are shown on a logarithmic scale. PSMA-RADS = prostate-specific membrane antigen reporting and data system.

Figure 2 displays SUV_max_ and SUV_peak_ among the different PSMA-RADS categories and organ compartments.

### 3.3. SUV_mean_ and Volumetric Parameters of PSMA-RADS-5 Lesions Were Susceptible to MIT Changes of 40 to 50%, But Not 40 to 45% or 45 to 50%

**Figure 3:**
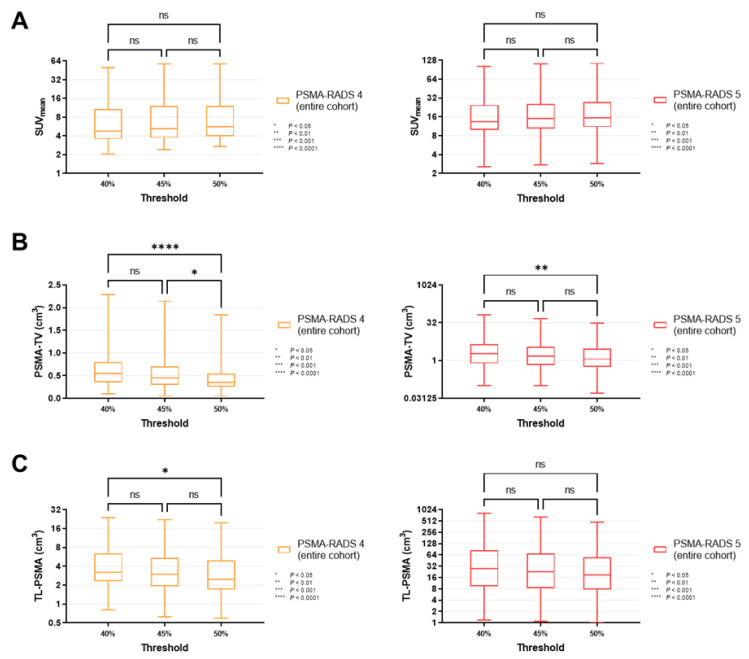
Box-and-whisker charts showing the mean standardized uptake values (SUV_mean_, (**A**)) and volumetric parameters [PSMA-avid tumor volume (PSMA-TV, (**B**)), and total lesion (TL-PSMA, (**C**))], depending on the chosen intensity threshold (40 vs. 45 vs. 50%), for all lesions classified as PSMA-RADS-4 or -5 (overall lesion level). (**A**) SUV_mean_ did not differ significantly between different intensity thresholds, for both PSMA-RADS-4 (*p* ≥ 0.34) and -5 lesions (*p* ≥ 0.30). (**B**) PSMA-TV changed significantly when changing the intensity threshold from 40 to 50% (PSMA-RADS-4, *p* < 0.0001; PSMA-RADS-5, *p* = 0.0066). When changing the intensity threshold in a smaller increment to 45%, the differences became largely insignificant (except for PSMA-RADS-4, *p* = 0.0450) (**C**) While changing the intensity threshold from 40 to 50% led to a significant difference in TL-PSMA of PSMA-RADS-4 lesions (*p* = 0.0272), TL-PSMA did not change significantly when smaller increments of 5% were applied (*p* ≥ 0.54). For PSMA-RADS-5 lesions, there was no statistical difference between the individual subgroups (*p* ≥ 0.16). Data are shown on a logarithmic scale (exception: linear scale for PSMA-TV in the PSMA-RADS-4 cohort). PSMA-RADS = prostate-specific membrane antigen reporting and data system.

**Figure 4:**
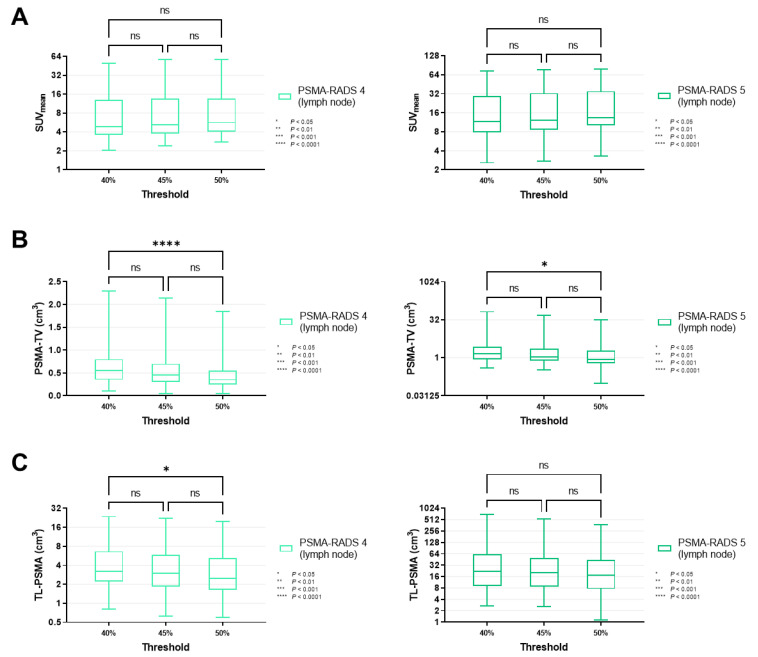
Box-and-whisker charts showing the mean standardized uptake values (SUV_mean_, (**A**)) and volumetric parameters [PSMA-avid tumor volume (PSMA-TV, (**B**)), and total lesion (TL-PSMA, (**C**))], depending on the chosen intensity threshold (40 vs. 45 vs. 50%), for lymph node lesions classified as PSMA-RADS-4 or -5. (**A**) Similar to SUV_mean_ on an overall lesion level (Figure 3A), values of lymph nodes categorized as PSMA-RADS-4 (*p* ≥ 0.40) and -5 (*p* ≥ 0.87) did not differ significantly between different intensity thresholds. (**B**,**C**) PSMA-TV reached significance in a comparison of intensity thresholds of 40 vs. 50% (*p* = 0.0239), in a manner similar to an overall lesion level (Figure 3B), thereby suggesting that these findings are primarily driven by lymph node lesions. The same applies to TL-PSMA (Figure 3C), which only showed a significant difference when comparing 40 and 50% in PSMA-RADS-4 lymph node lesions (*p* = 0.0453), but not for the skeleton (*p* ≥ 0.33, Figure 5). Data are shown on a logarithmic scale (exception: linear scale for PSMA-TV in the PSMA-RADS-4 cohort). PSMA-RADS = prostate-specific membrane antigen reporting and data system.

**Figure 5:**
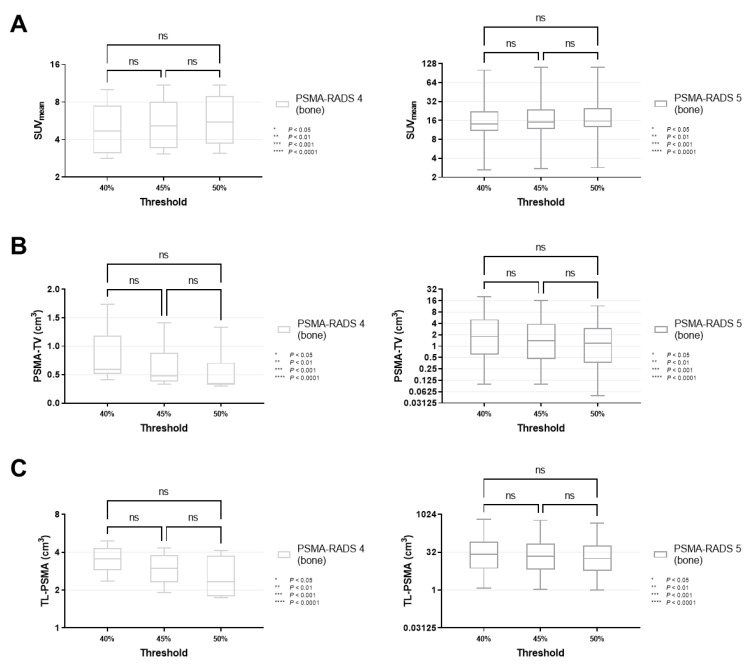
Box-and-whisker charts showing the mean standardized uptake values (SUV_mean_, (**A**)) and volumetric parameters [PSMA-avid tumor volume (PSMA-TV, (**B**)), and total lesion (TL-PSMA, (**C**))], depending on the chosen intensity threshold (40 vs. 45 vs. 50%), for bone lesions classified as PSMA-RADS-4 or -5. SUV_mean_ (*p* ≥ 0.31), PSMA-TV (*p* ≥ 0.11) and TL-PSMA (*p* ≥ 0.33) did not differ significantly when changing the intensity threshold. Data are shown on a logarithmic scale (exception: linear scale for PSMA-TV in the PSMA-RADS-4 cohort). PSMA-RADS = prostate-specific membrane antigen reporting and data system.

For all lesions classified as PSMA-RADS-4 (*p* ≥ 0.34) and -5 (*p* ≥ 0.30), SUV_mean_ did not change significantly when different MITs were applied (40 vs. 45 vs. 50%, Figure 3A). Similar results were recorded for PSMA-RADS-4/-5 lesions in the lymph nodes and skeleton (lymph nodes, *p* ≥ 0.40; skeleton, *p* ≥ 0.31, Figure 4A and Figure 5A).

By contrast, PSMA-TV differed significantly in both cohorts when comparing a MIT of 40 vs. 50% (PSMA-RADS-4, *p* < 0.0001; PSMA-RADS-5, *p* = 0.0066). When smaller increments of 5% were applied, there was still a significant difference in the PSMA-RADS-4 cohort for MIT of 45 vs. 50% (*p* = 0.0450). However, no significant level of difference was reached for MIT of 45 vs. 50% in the PSMA-RADS-5 cohort (*p* = 0.44) as well as for MIT of 40 vs. 45% in both cohorts (PSMA-RADS-4, *p* = 0.07; PSMA-RADS-5, *p* = 0.32, Figure 3B). On an organ-compartment level, those findings were primarily driven by lymph nodes (40 vs. 50% for PSMA-RADS-4, *p* < 0.0001; 40 vs. 50% for PSMA-RADS-5, *p* = 0.0239, Figure 4B), while PSMA-TV did not differ significantly in bone lesions (40 vs. 50% for PSMA-RADS-4, *p* = 0.11; 40 vs. 50% for PSMA-RADS-5, *p* = 0.15, Figure 5B). Again, when comparing smaller MIT differences, no significant difference of PSMA-TV was seen for lymph nodes (40 vs. 45% for PSMA-RADS-4, *p* = 0.10; 45 vs. 50% for PSMA-RADS-4, *p* = 0.07; 40 vs. 45% for PSMA-RADS-5, *p* = 0.72; 45 vs. 50% for PSMA-RADS-5, *p* = 0.42, Figure 4B).

For TL-PSMA, a significant difference was observed between MIT of 40 vs. 50% for PSMA-RADS-4 on an overall lesion level (*p* = 0.0272), whereas significance was not reached when changing the MIT to 45% (40 vs. 45% for PSMA-RADS-4, *p* = 0.61; 45 vs. 50% for PSMA-RADS-4, *p* = 0.54, Figure 3C). This was also driven by lymph nodes (40 vs. 45% for PSMA-RADS-4, *p* = 0.0453, Figure 4C). Regardless which MIT was applied, there were no significant differences of TL-PSMA in the PSMA-RADS-5 category, neither for all lesions (*p* ≥ 0.16, Figure 3C), nor for lymph node (*p* ≥ 0.67, Figure 4C) or bone lesions (*p* ≥ 0.56, Figure 5C).

**Figure 6:**
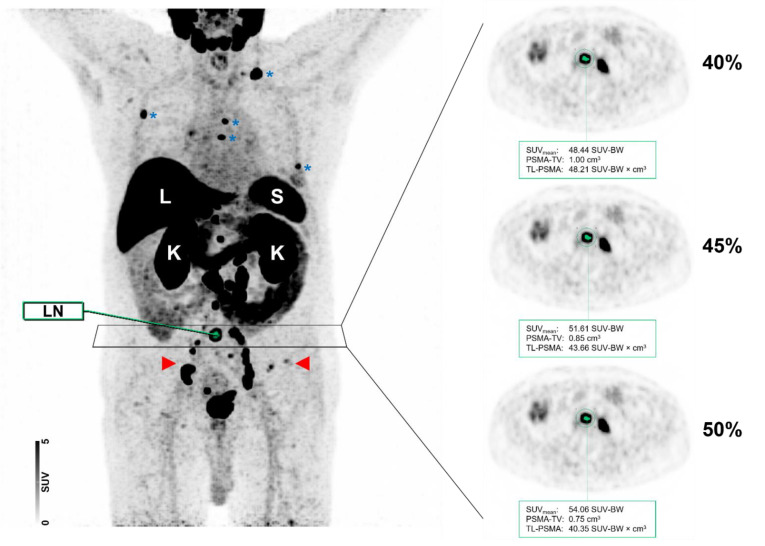
Maximum intensity projection (MIP) and axial positron emission tomography (PET) images of the same patient with different intensity thresholds (40 vs. 45 vs. 50%). Normal organ uptake in the liver (L), spleen (S), and kidneys (K) are indicated. The patient showed a relatively high tumor burden with multiple metastases involving both lymph nodes (red arrows) and the skeleton (blue asterisks). As shown for a PSMA-RADS-5 classified common iliac lymph node (LN), the segmentation did not differ visually when different maximum intensity thresholds of 40, 45, or 50% were applied.

Figure 6 displays a case of lymph node and bone involvement with different MIT, demonstrating no significant differences in the segmentation of a lymph node metastasis attributable to PC.

## 4. Discussion

In the present study, we aimed to evaluate semiquantitative parameters based on the PSMA-RADS scoring scheme by using the ^18^F-labeled PSMA-targeted imaging agent ^18^F-PSMA-1007. First, investigating PSMA-RADS-5 lesions, we revealed a significant difference of SUV_peak_ relative to PSMA-RADS-1A, -1B, -2, -3A, -3B, -3D, and -4, but not for -3C lesions. Of note, SUV_max_ demonstrated significant difference among all PSMA-RADS categories when compared to PSMA-RADS-5, and as such, both SUV parameters may be used in the context of a RADS-based scoring scheme [10,11].

Recent years have provided an increasing body of evidence of multiple pitfalls in the context of PSMA-targeted scan interpretation [23]. Nonetheless, given its high accuracy for staging of patients with PC, the number of conducted PET/CTs is expected to further grow, emphasizing the need for harmonized interpretation criteria that will allow the reader to identify lesions that are most likely attributable to PC. In this regard, several groups have made proposals for a structured interpretation system of PSMA-ligand PET/CT, including PROMISE, E-PSMA, and PSMA-RADS [10,11,24,25]. The latter system has already been applied to ^68^Ga-PSMA-11 and other ^18^F-labeled compounds, such as DCFPyL [14,26]. A comprehensive evaluation of PSMA-RADS for ^18^F-PSMA-1007 PET/CT, however, is still lacking. As such, in this study investigating patients with newly diagnosed, histologically proven and treatment-naïve PC at time of scan, we observed a high prevalence of lesions with intense uptake (PSMA-RADS-4 and -5, 39.5%) as well as of lesions with equivocal uptake (PSMA-RADS-3A or -3B, 20.9%) [27]. Relative to ^18^F-DCFPyL, the number of equivocal findings seems to be higher for ^18^F-PSMA-1007, in particular for the skeleton, which may partially explain the increased number of indeterminate PSMA-RADS-3A and PSMA-RADS-3B lesions observed in the present study [16]. Similar to ^18^F-DCFPyL [27], lymph nodes accounted for 449 of the 623 investigated lesions (72.1%), with the largest portion being either categorized as PSMA-RADS-2 or PSMA-RADS-3A. For ^18^F-DCFPyL, longitudinal studies have already been conducted to determine the true nature of such lesions, and PSMA-RADS-3A lesions (defined as equivocal uptake in soft tissue lesions) are more likely than RADS-3B (equivocal uptake in the bone) to represent true disease involvement [12].

In this study, we could prove that all benign lesions according to the PSMA-RADS system (categories PSMA-RADS-1A, -1B and -2) and lesions without relevant uptake (category PSMA-RADS-3D) had lower SUV_max_ and SUV_peak_ when compared with PSMA-RADS-5. For suspicious lesions of nonprostatic origin with high uptake (category PSMA-RADS-3C) we were also able to show a significant difference of SUV_max_ compared to PSMA-RADS-5.

The clinical most challenging lesions, i.e., equivocal lesions in categories PSMA-RADS-3A and -3B, had lower SUV_max_ and SUV_peak_ when compared with PSMA-RADS-5. Therefore, we are convinced that an analysis of SUV based on PSMA-RADS may be helpful for stratifying the risk of PC involvement, in particular for lymph nodes and for the discrimination of equivocal PSMA-RADS-3B lesions in the bone. Lesions in category PSMA-RADS-3A, moreover, had a significantly lower SUV_max_ and SUV_peak_ compared to PSMA-RADS-4 lesions, while PSMA-RADS-3B lesions did not. This might be due to the small number of PSMA-RADS-4 lesions in the bone, and we believe that this may become significant in a larger cohort. SUV_max_ and SUV_peak_ of PSMA-RADS-4 bone lesions did not significantly differ from the entire PSMA-RADS-5 cohort, which proves the concept of the PSMA-RADS scoring scheme. Of note, findings of lesion distribution among different PSMA-RADS categories and SUVs are in line with a recent observation of Ashrafinia et al. using ^18^F-DCFPyL, supporting the notion that semi-quantification based on the PSMA-RADS scoring scheme can be applied to a broad spectrum of ^18^F-labeled PSMA agents [27].

In addition, the SUVs may also be helpful for the interpreting molecular imaging specialist by assigning the appropriate PSMA-RADS score to a suspicious lesion, thereby increasing diagnostic certainty. Identifying patients with low-volume disease, i.e., with less than four bone metastases, is of high therapeutic relevance because these patients benefit from multimodal therapy, as the CHAARTED (Chemohormonal Therapy Versus Androgen Ablation Randomized Trial for Extensive Disease in Prostate Cancer) trial has shown [28]. Furthermore, the results on SUV may be of importance in the context of response assessment. For instance, using the ^18^F-labeled radiotracer DCFPyL, Zukotynski et al. investigated men with castration-resistant PC prior to abiraterone or enzalutamide treatment. All patients were imaged prior to therapy and 2 to 4 months during follow-up. The authors used delta percent (DPSM) and delta absolute SUV_max_ (DASM), which represent the changes in radiotracer accumulation between both scans. Increased DPSM/DASM was linked with poorer overall survival [29]. Given significant differences of SUV_max_ in PSMA-RADS-5 lesions relative to other lesions along with the herein presented SUV_max_ values for this category, the reader may have certainty that relevant lesions for such response assessments will not be missed. Such considerations on SUV_max_ may also be relevant to identify treatment responders in patients scheduled for PSMA-directed radioligand therapy, as baseline SUV_max_ has been advocated to identify high-risk individuals [30,31,32]. Those studies, however, have been conducted using ^68^Ga-labeled radiotracers, whereas for ^18^F-PSMA-1007, such investigations are still needed, which may further refine the prognostic value of SUV_max_ for treatment failure [30,33].

SUV_mean_ and volumetric parameters, i.e., PSMA-TV and TL-PSMA, can be influenced by different MITs, and as such, different thresholds (40 vs. 45 vs. 50%) have been applied to conduct a semi-quantitative analysis of the PSMA-avid tumor burden at baseline. The size of the VOI, however, was not changed for a given lesion. For all PSMA-RADS-5 classified lesions, SUV_mean_ and TL-PSMA were not influenced by changing the MIT from 40 to 45 or 50%. For PSMA-TV, significance was reached when comparing 40 vs. 50% MIT, but not when incremental steps of 5% were used. The same phenomenon applied for lymph nodes, while PSMA-TV of bone lesions was not affected by changes of the MIT, suggesting lymph nodes are more prone to threshold alterations. Of note, level of significance regarding TL-PSMA was only reached for 10% changes of the intensity threshold in PSMA-RADS-4 lesions (overall lesion and lymph node level). For SUV_mean_ and bone lesions in general, no significant differences were observed for varying the MIT between 40, 45 and 50% in PSMA-RADS-4/-5 lesions. Again, such findings may be of importance for response assessments. The recently proposed PSMA PET Progression Criteria suggested an increase in tracer uptake by ≥30% to be indicative for progressive disease. The authors did not recommend a certain semiquantitative parameter for this approach, but findings of the present study may indicate that a MIT of 40–50% might be suitable, regardless of whether volumetric parameters or SUV_mean_ will be used for this purpose [34]. Taken together, in particular for lymph nodes a threshold of 45% may be considered as safe to provide reliable results.

This study has several limitations. Our retrospective cohort includes a small number of subjects and thus, our preliminary results warrant further confirmation in a prospective study. Second, histopathological confirmation of lesions would be desirable in the context of PSMA-RADS, but is not feasible. Given the high prevalence of PSMA-RADS-3A lesions in the present study, longitudinal studies in the context of ^18^F-PSMA-1007 would also be needed, as information on the true nature of those findings may guide therapy [13]. Third, the herein presented findings may primarily apply to patients referred for initial staging. As such, semiquantitative values, along with lesion distribution across different PSMA-RADS scores, may differ in other cohorts, e.g., in patients scheduled for PSMA-directed radioligand therapy [35]. Furthermore, even though the reconstruction algorithm was conducted as implemented by the manufacturer, the results on scanning parameters for lymph nodes are also susceptible to different scanning protocols, which makes inter-institutional comparisons difficult. Although our results seem to be comparable to other ^18^F-labeled radiotracers [27], further studies investigating existing and novel PSMA-directed compounds are needed, including ^68^Ga-PSMA-11.

## 5. Conclusions

Semiquantitative characterization of the structured reporting system PSMA-RADS in patients imaged with ^18^F-PSMA-1007 PET/CT revealed that SUV_max_ was significantly higher in PSMA-RADS-5 classified lesions compared to all other PSMA-RADS categories. As such, the latter parameter may assist the interpreting molecular imaging specialist to assign the correct PSMA-RADS score to sites of disease, thereby increasing diagnostic certainty. In addition, changes of the MIT in PSMA-RADS-5 lesions had no significant impact on SUV_mean_ and TL-PSMA in contrast to PSMA-TV.

## Figures and Tables

**Table 1:** Characteristics of the study population.

Parameter	Value
Subjects	18
Age (y)	
Mean ± SD	70 ± 8
Range	50–80
PSA (ng/mL)	
Mean ± SD	180.35 ± 393.75
Range	4.79–1690.00
Gleason score	
Median	8
Range	7–10

SD = standard deviation, PSA = prostate-specific antigen.

## Data Availability

The data presented in this study are available in the article and in Appendix A.

## Appendix A

**Table A1:** Descriptive statistics of intensity of tracer uptake and volumetric ^18^F-PSMA PET/CT parameters of all lesions separated by PSMA-RADS category and compartment.

PSMA-RADS Category & Compartment	Parameter	Threshold	Mean ± SD	Range
1A—Lung	SUV_max_		1.07 ± 0.50	0.54–1.72
	SUV_peak_		0.83 ± 0.26	0.57–1.13
	SUV_mean_	40%	0.80 ± 0.29	0.49–1.18
		45%	0.80 ± 0.29	0.49–1.19
		50%	0.83 ± 0.32	0.49–1.24
	PSMA-TV	40%	0.51 ± 0.46	0.15–1.34
		45%	0.50 ± 0.44	0.15–1.29
		50%	0.45 ± 0.39	0.15–1.14
	TL-PSMA	40%	0.50 ± 0.61	0.08–1.58
		45%	0.49 ± 0.59	0.08–1.54
		50%	0.45 ± 0.54	0.08–1.42
1B—Bone	SUV_max_		3.49 ± 1.08	2.73–4.26
	SUV_peak_		2.47 ± 0.64	2.02–2.93
	SUV_mean_	40%	2.20 ± 0.77	1.66–2.75
		45%	2.40 ± 0.82	1.82–2.98
		50%	2.49 ± 0.82	1.91–3.08
	PSMA-TV	40%	2.16 ± 0.53	1.79–2.54
		45%	1.69 ± 0.35	1.44–1.94
		50%	1.46 ± 0.24	1.29–1.64
	TL-PSMA	40%	4.57 ± 0.50	4.22–4.93
		45%	3.90 ± 0.54	3.52–4.29
		50%	3.56 ± 0.60	3.14–3.99
1B—Lymph Node	SUV_max_		1.46 ± 0.50	0.69–3.33
	SUV_peak_		0.87 ± 0.27	0.54–1.93
	SUV_mean_	40%	1.02 ± 0.36	0.48–2.16
		45%	1.05 ± 0.37	0.48–2.25
		50%	1.09 ± 0.39	0.48–2.47
	PSMA-TV	40%	0.67 ± 0.57	0.10–3.09
		45%	0.61 ± 0.48	0.10–2.41
		50%	0.53 ± 0.39	0.10–2.07
	TL-PSMA	40%	0.64 ± 0.53	0.10–2.84
		45%	0.60 ± 0.48	0.10–2.67
		50%	0.55 ± 0.43	0.10–2.36
1B—Liver	SUV_max_		11.05 ± 1.96	9.13–13.06
	SUV_peak_		10.62 ± 1.70	8.73–12.05
	SUV_mean_	40%	10.42 ± 1.76	8.93–12.37
		45%	10.42 ± 1.76	8.93–12.37
		50%	10.42 ± 1.76	8.93–12.37
	PSMA-TV	40%	0.20 ± 0.04	0.17–0.25
		45%	0.20 ± 0.04	0.17–0.25
		50%	0.20 ± 0.04	0.17–0.25
	TL-PSMA	40%	2.14 ± 0.57	1.48–2.48
		45%	2.14 ± 0.57	1.48–2.48
		50%	2.14 ± 0.57	1.48–2.48
1B—Lung	SUV_max_		1.40 ± 0.00	1.40–1.40
	SUV_peak_		1.29 ± 0.00	1.29–1.29
	SUV_mean_	40%	1.25 ± 0.00	1.25–1.25
		45%	1.25 ± 0.00	1.25–1.25
		50%	1.25 ± 0.00	1.25–1.25
	PSMA-TV	40%	0.33 ± 0.00	0.33–0.33
		45%	0.33 ± 0.00	0.33–0.33
		50%	0.33 ± 0.00	0.33–0.33
	TL-PSMA	40%	0.42 ± 0.00	0.42–0.42
		45%	0.42 ± 0.00	0.42–0.42
		50%	0.42 ± 0.00	0.42–0.42
1B—Soft Tissue	SUV_max_		9.14 ± 5.63	1.76–21.67
	SUV_peak_		8.54 ± 4.80	1.05–15.86
	SUV_mean_	40%	6.30 ± 4.36	1.01–15.87
		45%	6.63 ± 4.34	1.08–15.87
		50%	7.07 ± 4.62	1.16–17.04
	PSMA-TV	40%	2.26 ± 2.96	0.10–7.96
		45%	1.73 ± 2.22	0.10–6.39
		50%	1.33 ± 1.66	0.10–4.81
	TL-PSMA	40%	6.11 ± 4.01	1.05–16.14
		45%	5.14 ± 3.01	1.05–11.96
		50%	4.36 ± 2.31	0.76–9.09
2—Lymph Node	SUV_max_		3.13 ± 1.18	1.03–7.52
	SUV_peak_		1.88 ± 0.70	0.54–3.83
	SUV_mean_	40%	2.09 ± 0.72	0.69–4.12
		45%	2.17 ± 0.75	0.71–4.42
		50%	2.27 ± 0.81	0.74–5.03
	PSMA-TV	40%	0.89 ± 0.69	0.15–3.68
		45%	0.78 ± 0.59	0.15–3.28
		50%	0.67 ± 0.49	0.08–2.44
	TL-PSMA	40%	1.90 ± 1.83	0.26–9.45
		45%	1.74 ± 1.63	0.26–8.73
		50%	1.55 ± 1.41	0.21–7.05
3A—Lymph Node	SUV_max_		3.28 ± 0.97	1.40–8.58
	SUV_peak_		1.90 ± 0.62	0.89–4.47
	SUV_mean_	40%	2.09 ± 0.60	0.97–5.03
		45%	2.22 ± 0.64	1.04–5.41
		50%	2.33 ± 0.66	1.05–5.62
	PSMA-TV	40%	1.03 ± 0.72	0.17–4.73
		45%	0.85 ± 0.61	0.15–4.13
		50%	0.72 ± 0.50	0.15–3.48
	TL-PSMA	40%	2.11 ± 1.53	0.35–10.79
		45%	1.85 ± 1.36	0.31–9.81
		50%	1.63 ± 1.18	0.31–8.65
3B—Bone	SUV_max_		4.78 ± 0.78	3.11–5.88
	SUV_peak_		2.37 ± 0.31	1.86–3.29
	SUV_mean_	40%	2.85 ± 0.51	1.73–3.67
		45%	3.08 ± 0.53	2.06–4.00
		50%	3.29 ± 0.55	2.20–4.20
	PSMA-TV	40%	1.38 ± 1.13	0.41–5.62
		45%	1.03 ± 0.73	0.33–3.73
		50%	0.80 ± 0.57	0.25–3.04
	TL-PSMA	40%	3.61 ± 2.42	1.41–12.94
		45%	2.97 ± 1.77	1.05–9.54
		50%	2.48 ± 1.50	0.86–8.13
3C—Lymph Node	SUV_max_		5.09 ± 0.82	4.37–6.63
	SUV_peak_		3.66 ± 1.89	1.76–6.39
	SUV_mean_	40%	3.11 ± 0.52	2.69–3.95
		45%	3.36 ± 0.52	2.94–4.31
		50%	3.59 ± 0.64	3.05–4.75
	PSMA-TV	40%	1.46 ± 0.80	0.35–2.59
		45%	1.18 ± 0.70	0.25–2.19
		50%	0.95 ± 0.56	0.25–1.69
	TL-PSMA	40%	4.61 ± 2.64	0.97–7.46
		45%	4.01 ± 2.37	0.79–6.45
		50%	3.42 ± 1.99	0.79–5.30
3C—Lung	SUV_max_		5.15 ± 0.00	5.15–5.15
	SUV_peak_		2.79 ± 0.00	2.79–2.79
	SUV_mean_	40%	3.27 ± 0.00	3.27–3.27
		45%	3.44 ± 0.00	3.44–3.44
		50%	3.51 ± 0.00	3.51–3.51
	PSMA-TV	40%	0.75 ± 0.00	0.75–0.75
		45%	0.65 ± 0.00	0.65–0.65
		50%	0.60 ± 0.00	0.60–0.60
	TL-PSMA	40%	2.44 ± 0.00	2.44–2.44
		45%	2.22 ± 0.00	2.22–2.22
		50%	2.10 ± 0.00	2.10–2.10
3C—Soft Tissue	SUV_max_		5.78 ± 1.05	4.65–7.19
	SUV_peak_		4.02 ± 1.54	2.75–6.25
	SUV_mean_	40%	3.62 ± 1.37	2.65–5.66
		45%	3.87 ± 1.46	2.84–6.04
		50%	4.07 ± 1.32	3.18–6.04
	PSMA-TV	40%	4.73 ± 4.10	0.40–9.21
		45%	3.60 ± 3.03	0.35–6.62
		50%	2.61 ± 2.18	0.35–4.53
	TL-PSMA	40%	14.54 ± 12.28	2.25–27.06
		45%	11.92 ± 9.77	2.11–20.96
		50%	9.33 ± 7.49	2.11–16.12
3D—Lymph Node	SUV_max_		2.48 ± 0.86	1.54–3.24
	SUV_peak_		2.04 ± 0.47	1.65–2.57
	SUV_mean_	40%	1.56 ± 0.39	1.11–1.81
		45%	1.61 ± 0.45	1.11–1.97
		50%	1.70 ± 0.53	1.11–2.14
	PSMA-TV	40%	1.69 ± 1.55	0.66–3.48
		45%	1.38 ± 1.01	0.66–2.54
		50%	1.12 ± 0.64	0.58–1.84
	TL-PSMA	40%	2.84 ± 3.00	1.05–6.31
		45%	2.41 ± 2.25	1.05–5.01
		50%	2.02 ± 1.66	1.05–3.94
3D—Lung	SUV_max_		0.89 ± 0.55	0.42–2.54
	SUV_peak_		0.79 ± 0.38	0.38–1.63
	SUV_mean_	40%	0.74 ± 0.39	0.39–1.76
		45%	0.76 ± 0.44	0.39–2.11
		50%	0.76 ± 0.44	0.39–2.11
	PSMA-TV	40%	0.26 ± 0.14	0.08–0.60
		45%	0.26 ± 0.14	0.08–0.60
		50%	0.25 ± 0.13	0.08–0.55
	TL-PSMA	40%	0.20 ± 0.16	0.04–0.68
		45%	0.20 ± 0.16	0.04–0.68
		50%	0.20 ± 0.16	0.04–0.64
3D—Soft Tissue	SUV_max_		1.79 ± 0.00	1.79–1.79
	SUV_peak_		1.04 ± 0.00	1.04–1.04
	SUV_mean_	40%	1.15 ± 0.00	1.15–1.15
		45%	1.23 ± 0.00	1.23–1.23
		50%	1.32 ± 0.00	1.32–1.32
	PSMA-TV	40%	0.60 ± 0.00	0.60–0.60
		45%	0.50 ± 0.00	0.50–0.50
		50%	0.40 ± 0.00	0.40–0.40
	TL-PSMA	40%	0.69 ± 0.00	0.69–0.69
		45%	0.61 ± 0.00	0.61–0.61
		50%	0.53 ± 0.00	0.53–0.53
4—Bone	SUV_max_		8.55 ± 3.76	4.49–14.98
	SUV_peak_		3.44 ± 1.02	2.47–5.27
	SUV_mean_	40%	5.35 ± 2.67	2.83–10.02
		45%	5.82 ± 2.90	3.06–10.99
		50%	6.22 ± 2.94	3.11–10.99
	PSMA-TV	40%	0.81 ± 0.49	0.41–1.74
		45%	0.63 ± 0.40	0.33–1.41
		50%	0.52 ± 0.40	0.30–1.33
	TL-PSMA	40%	3.59 ± 0.91	2.36–4.93
		45%	3.05 ± 0.91	1.91–4.31
		50%	2.66 ± 1.02	1.74–4.13
4—Lymph Node	SUV_max_		15.64 ± 15.98	3.13–74.57
	SUV_peak_		6.08 ± 5.72	1.43–33.17
	SUV_mean_	40%	10.24 ± 10.83	2.04–49.97
		45%	11.09 ± 11.81	2.40–57.06
		50%	11.77 ± 12.60	2.72–57.33
	PSMA-TV	40%	0.64 ± 0.42	0.10–2.29
		45%	0.53 ± 0.35	0.05–2.14
		50%	0.42 ± 0.27	0.05–1.84
	TL-PSMA	40%	4.89 ± 4.06	0.81–23.55
		45%	4.30 ± 3.69	0.62–22.35
		50%	3.73 ± 3.19	0.59–19.59
5—Bone	SUV_max_		30.27 ± 25.79	4.28–157.80
	SUV_peak_		15.26 ± 12.58	1.67–77.70
	SUV_mean_	40%	18.84 ± 16.47	2.63–101.10
		45%	20.25 ± 17.55	2.73–112.90
		50%	21.70 ± 19.52	2.84–113.10
	PSMA-TV	40%	3.55 ± 4.52	0.10–20.60
		45%	2.83 ± 3.55	0.10–16.12
		50%	2.28 ± 2.79	0.05–11.59
	TL-PSMA	40%	69.29 ± 106.80	1.18–662.10
		45%	59.66 ± 93.39	1.09–598.00
		50%	50.35 ± 76.10	0.99–463.60
5—Lymph Node	SUV_max_		34.12 ± 28.88	4.63–120.30
	SUV_peak_		18.05 ± 14.82	2.81–58.54
	SUV_mean_	40%	21.00 ± 18.13	2.57–73.98
		45%	22.35 ± 19.15	2.73–76.67
		50%	23.73 ± 20.05	3.28–79.29
	PSMA-TV	40%	4.13 ± 11.00	0.40–66.09
		45%	3.11 ± 7.62	0.33–46.88
		50%	2.26 ± 4.99	0.10–31.15
	TL-PSMA	40%	65.12 ± 127.00	2.72–695.90
		45%	53.78 ± 96.57	2.57–529.60
		50%	43.32 ± 70.85	1.13–377.30
5—Soft Tissue	SUV_max_		32.32 ± 8.81	23.32–40.93
	SUV_peak_		15.12 ± 10.32	8.15–26.98
	SUV_mean_	40%	20.15 ± 3.57	16.65–23.79
		45%	21.27 ± 4.13	16.65–24.63
		50%	20.20 ± 16.31	5.95–37.99
	PSMA-TV	40%	0.95 ± 1.06	0.33–2.19
		45%	0.84 ± 0.95	0.25–1.94
		50%	1.50 ± 1.20	0.33–2.74
	TL-PSMA	40%	21.52 ± 26.47	5.52–52.08
		45%	19.64 ± 24.38	5.52–47.79
		50%	25.54 ± 25.92	5.52–54.82
5—Primary	SUV_max_		32.66 ± 21.91	5.15–82.80
	SUV_peak_		18.85 ± 12.52	3.58–50.20
	SUV_mean_	40%	18.13 ± 13.27	2.89–49.96
		45%	20.01 ± 14.69	3.10–53.51
		50%	21.37 ± 15.21	3.33–56.03
	PSMA-TV	40%	9.16 ± 8.38	0.25–28.96
		45%	6.36 ± 6.09	0.17–20.70
		50%	4.32 ± 4.20	0.17–14.68
	TL-PSMA	40%	145.90 ± 192.80	10.59–806.90
		45%	111.50 ± 154.00	8.22–652.00
		50%	82.76 ± 112.60	5.36–472.30

SD = standard deviation, SUV_max_ = maximum standardized uptake value corrected to body weight, SUV_peak_ = peak standardized uptake value corrected to body weight, SUV_mean_ = mean standardized uptake value corrected to body weight, PSMA-TV = PSMA-derived tumor volume (cm^3^), TL-PSMA = total lesion PSMA (cm^3^).

**Table A2:** Descriptive statistics of number of lesions per PSMA-RADS category and total lesions for each of the 18 patients.

PSMA-RADS Category	Pat. #1	Pat. #2	Pat. #3	Pat. #4	Pat. #5	Pat. #6	Pat. #7	Pat. #8	Pat. #9
1A	0	1	0	0	1	0	1	0	0
1B	3	0	1	3	7	3	4	1	0
2	4	8	3	10	9	7	22	1	13
3A	0	13	1	7	14	14	6	3	1
3B	0	9	0	2	1	2	0	0	1
3C	0	3	0	0	0	0	0	0	0
3D	0	0	0	0	0	1	0	1	5
4	10	12	2	1	4	3	3	1	0
5	25	9	1	1	1	8	5	1	2
Total	42	55	8	24	37	38	41	8	22
**PSMA-RADS Category**	**Pat. #10**	**Pat. #11**	**Pat. #12**	**Pat. #13**	**Pat. #14**	**Pat. #15**	**Pat. #16**	**Pat. #17**	**Pat. #18**
1A	1	0	0	0	0	0	0	0	1
1B	6	12	11	22	2	6	1	2	1
2	13	6	7	6	2	6	0	1	3
3A	7	9	1	7	1	2	0	3	16
3B	3	0	0	0	3	2	0	0	2
3C	0	2	0	0	0	0	0	0	6
3D	1	1	0	1	9	0	1	1	4
4	9	4	3	19	3	14	0	3	7
5	2	15	15	15	23	21	2	1	1
Total	42	49	37	70	43	51	4	11	41

Pat. = patient.
